# Evaluation of Antioxidant and Immunity Function of Tetramethylpyrazine Phosphate Tablets *in Vivo*

**DOI:** 10.3390/molecules17055412

**Published:** 2012-05-08

**Authors:** Ling Guo, Aihong Wang, Yongle Sun, Chongen Xu

**Affiliations:** 1 Department of Cardiology, Provincial Hospital Affiliated to Shandong University, 324# Jingwu-Weiqi Road, Jinan 250021, China; Email: lguodrjn@163.com (L.G.); ahwangdf@163.com(A.W.); ylsunfjh@163.com(Y.S.); 2 Department of Cardiac Surgery, Provincial Hospital Affiliated to Shandong University, 324# Jingwu-Weiqi Road, Jinan 250021, China

**Keywords:** tetramethylpyrazine phosphate tablets, LVEDP, LVSP, GSH-Px

## Abstract

The aim of the study was to determine the effect of tetramethylpyrazine phosphate tablets (TPT), a Chinese medicine used for cardiovascular disease, on immunity activity and oxidative injury in rats. Heart failure (HF) was induced by isoproterenol (ISO). After the animal model was established, the rats were administered the TPT by gavage (once a day). The results indicated that TPT improved left ventricular systolic pressure (LVSP), left ventricular end-diastolic pressure (LVEDP), ±dP/dt, heart weight/body weight. TPT could decrease the levels of tumor necrosis factor-α (TNF-α) and interleukin 6 (IL-6). Furthermore, it also could raise the activities of catalase, superoxide dismutase (SOD) and glutathione peroxidase (GSH-Px), but reduce malonyldialdehyde (MDA) level. The results indicated that TPT improved cardiac function and myocardial fibrosis from myocardial injury, and this cardioprotection might be attributed to a reduction of oxidative stress and regulation of inflammation mediators.

## 1. Introduction

Heart failure is rising in prevalence, and is associated with increased morbidity, repeat hospitalizations, high mortality rates and health care expenditures. It constitutes a severe burden of disease both on a personal and a societal level [1]. Major depression is 4–5 times more common in heart failure patients than in the general population [2]. In acute heart failure, such as in the setting of acute myocardial ischemia, or hypertensive crisis, dyspnea is directly related to abnormal hemodynamics. A chain of events beginning with cardiac dysfunction and decreased cardiac output, which in turn leads to neurohumoral activation, cytokine release, and decreased muscle blood flow [3,4].

Heart failure has been found to be associated with oxidative stress in animal studies, with a concomitant lowering in antioxidant enzyme activity [5,6,7,8], and with antioxidant depletion in plasma [8,9] and heart [5,6,7] in animal studies. Free radical production has been related in CHF to catecholamine autoxidation and recurrent episodes of ischemia and reperfusion [10]. Myocardial oxidative stress due to increased generation of reactive oxygen species (ROS) plays a recognized role in the pathogenesis as well as the progression of heart failure (HF) [11,12,13,14]. The myocardium is equipped with a variety of endogenous enzymatic and non-enzymatic antioxidant systems that are sufficient to metabolize ROS. Furthermore, ROS also stimulate signal transduction to elaborate inflammatory cytokines, e.g., tumor necrosis factor alpha (TNF-α) and interleukin-6 (IL-6), in the ischemic region and surrounding myocardium as a host reaction. It is becoming increasingly apparent that inflammatory mediators play a crucial role in the development of heart failure, and several strategies to counterbalance different aspects of the inflammatory response are considered [15].

Tetramethylpyrazine (TMP) is a biomonomer with molecular formula C_8_H_12_N_2_·HCl·2H_2_O extracted from the traditional Chinese herbal medicine Chuanxiong. Previous studies have shown the neuroprotective effects of TMP on cerebral ischemic damage in models of global [16,17] and focal [17,18,19] cerebral ischemia. TMP has been demonstrated to increase cerebral blood flow [20], improve microcirculation [21], and reduce capillary permeability [22]. Some of those studies have indicated that TMP reduces ischemia/reperfusiondamage via inhibition of HIF-1alpha [18], TNF-alpha activations [18], apoptosis formation [18], and free radical scavenger activity [17], suggesting that TMP reduces ischemia/reperfusion damage through multiple mechanisms.

Tetramethylpyrazine phosphate tablets is a Chinese medicine, which is widely used in clinical therapy of cardiovascular disease. In the present study, we examine effect of tetramethylpyrazine phosphate tablets on immunity and antioxidant enzymes activities in experimental rats.

## 2. Results

During ISO treatment, two rats died in the ISO model; two rats died in TPT+ISO (2 mg/kg); three rats in TPT+ISO (3 mg/kg); two rats died in TPT+ISO (4 mg/kg). As shown in Table 1, LVEDP was significantly (*p* < 0.001) enhanced, whereas LVSP, +dp/dtmax, and −dp/dtmax were significantly decreased in untreated ISO model rats in comparison to normal control. Treatment with 2, 3 and 4 mg/kg of the TPT dose-dependently significantly decreased the LVEDP, and increased LVSP, +dp/dtmax, and −dp/dtmax in TPT+ISO groups compared to the untreated ISO model group. 

**Table 1:** Effect of TPT on LVEDP, LVSP, +dp/dtmax, and −dp/dtmax in all groups of rats.

Group	Left ventricular end diastolic pressure (mmHg)	Left ventricular systolic pressure (mmHg)	+dp/dtmax (mmHg/s)	−dp/dtmax (mmHg/s)
Normal	5.61 ± 0.36	118.32 ± 12.09	3215.85 ± 276.83	3064.44 ± 274.13
ISO model	11.07 ± 0.96 **	99.26 ± 6.73 **	2087.59 ± 187.36 **	2007.46 ± 184.07 **
TPT+ISO (2 mg/kg)	8.21 ± 0.74 ^##^	105.32 ± 8.64	2548.65 ± 215.73 ^##^	2316.49 ± 198.67 ^##^
TPT+ISO (3 mg/kg)	7.05 ± 0.63 ^##^	107.43 ± 5.93	2749.06 ± 251.16 ^##^	2781.77 ± 201.74 ^##^
TPT+ISO (4 mg/kg)	6.11 ± 0.54 ^##^	113.65 ± 7.07 ^#^	3081.16 ± 255.78 ^##^	2991.83 ± 222.69 ^##^

** *p* < 0.01, ISO model group *vs*. normal control group; ^#^ *p* < 0.05, ^##^ *p* < 0.01, TPT+ISO groups *vs.* ISO model group.

Compared with the normal control group, rats in untreated ISO model group demonstrated a higher LVMI and RVMI (*p* < 0.001; Table 2). The extract with 2, 3 and 4 mg/kg of the TPT dose-dependently significantly reduced this elevation in TPT+ISO groups compared to the untreated ISO model group (*p* < 0.05; Table 2).

**Table 2:** Effect of TPT on LVMI and RVMI in all groups of rats.

Group	LVMI (mg/g)	RVMI (mg/g)
Normal	2.31 ± 0.22	0.65 ± 0.05
ISO model	3.57 ± 0.27 **	0.77 ± 0.05 **
TPT+ISO (2 mg/kg)	3.21 ± 0.24 ^#^	0.73 ± 0.06
TPT+ISO (3 mg/kg)	2.89 ± 0.21 ^##^	0.69 ± 0.05 ^#^
TPT+ISO (4 mg/kg)	2.53 ± 0.23 ^##^	0.66 ± 0.06 ^#^

** *p* < 0.01, ISO model group *vs.* normal control group; ^#^ *p* < 0.05, ^##^ *p* < 0.01, TPT+ISO groups *vs.* ISO model group.

Increased serum Ang II, cholesterol, ANP and endothelin levels of the untreated ISO model group were observed when compared with the normal control (Table 3). It is evident from our results (Table 3) that pretreatment with TPT significantly (*p* < 0.05) decreased Ang II, cholesterol, ANP and endothelin levels when compared with the untreated ISO model group. Moreover, the exhibited effect was dose dependent. 

The concentration of TNF-α and IL-6 was increased in untreated ISO model rats, but treatment with TPT decreased the TNF-α and IL-6 levels in TPT+ISO groups compared to the untreated ISO model group (Table 4).

Table 5 shows the activity of superoxide dismutase (SOD), GSH-Px and catalase (CAT) as well as MDA, GSH concentrations in myocardium tissue of all groups of rats. Untreated ISO model group rats showed a significant decrease in the activity of these enzymes (SOD, GSH-Px and CAT) and GSH, and a significant increase in the levels of MDA. Rats treated with TPT (2, 3 and 4 mg/kg) showed a significant increase in the activity of SOD, GSH and CAT, GSH as well as a significant decrease in the levels MDA in the myocardium tissue of the TPT-treated rats (TPT+ISO) when compared to untreated ISO model rats.

**Table 3:** Effect of TPT on serum Ang II, Cholesterol, ANP and Endothelin levels in all groups of rats.

Group	Ang II (pg/mL)	Cholesterol (ng/mL)	ANP (ng/mL)	Endothelin (pg/mL)
Normal	1408.32 ± 112.73	0.605 ± 0.046	0.687 ± 0.048	161.52 ± 11.75
ISO model	1676.81 ± 140.27 **	1.241 ± 0.094 **	1.085 ± 0.088 **	219.04 ± 15.48 **
TPT+ISO (2 mg/kg)	1582.09 ± 121.53 ^#^	1.042 ± 0.082 ^##^	0.913 ± 0.073 ^##^	201.16 ± 18.04 ^#^
TPT+ISO (3 mg/kg)	1503.17 ± 131.64 ^##^	0.831 ± 0.067 ^##^	0.831 ± 0.065 ^##^	188.42 ± 15.83 ^##^
TPT+ISO (4 mg/kg)	1437.83 ± 133.29 ^##^	0.683 ± 0.049 ^##^	0.715 ± 0.069 ^##^	170.47 ± 14.92 ^##^

** *p* < 0.01, ISO model group *vs*. normal control group; ^#^ *p* < 0.05, ^##^ *p* < 0.01, TPT+ISO groups *vs.* ISO model group.

**Table 4:** Effect of TPT on serum TNF-α and IL-6 levels in all groups of rats.

Group	TNF-α (ng/L)	IL-6 (ng/L)
Normal	61.74 ± 4.83	111.64 ± 10.75
ISO model	174.27 ± 12.15 **	241.47 ± 14.28 **
TPT+ISO (2 mg/kg)	142.68 ± 11.09 ^##^	203.62 ± 12.63 ^##^
TPT+ISO (3 mg/kg)	101.23 ± 7.69 ^##^	175.39 ± 11.49 ^##^
TPT+ISO (4 mg/kg)	75.29 ± 4.62 ^##^	136.18 ± 9.75 ^##^

** *p* < 0.01, ISO model group *vs.* normal control group; ^##^ *p* < 0.01, TPT+ISO groups *vs.* ISO model group.

**Table 5:** Effect of TPT on myocardium MDA and GSH levels, SOD, CAT and GSH-Px activities in all groups of rats.

Group	MDA	GSH	SOD	CAT	GSH-Px
Normal	4.05 ± 0.25	132.7 ± 12.6	254.4 ± 32.8	35.13 ± 2.96	42.71 ± 3.09
ISO model	7.49 ± 0.53 **	78.4 ± 4.6 **	127.3 ± 10.9 **	13.29 ± 1.76 **	21.69 ± 1.57 **
TPT+ISO (2 mg/kg)	6.11 ± 0.47 ^##^	94.1 ± 4.7 ^##^	164.8 ± 15.5 ^##^	18.95 ± 1.49 ^##^	28.46 ± 1.63 ^##^
TPT+ISO (3 mg/kg)	5.24 ± 0.32 ^##^	115.7 ± 10.7 ^##^	199.2 ± 20.8 ^##^	25.75 ± 1.83 ^##^	36.92 ± 1.71 ^##^
TPT+ISO (4 mg/kg)	4.32 ± 0.24 ^##^	142.6 ± 13.1 ^##^	248.1 ± 23.1 ^##^	33.86 ± 2.07 ^##^	40.62 ± 2.69 ^##^

** *p* < 0.01, ISO model group *vs*. normal control group; ^##^ *p* < 0.01, TPT+ISO groups *vs.* ISO model group.

Histopathological examination showed that normal control rats’ heart displayed normal architecture. Rats’ heart in the ISO-administered group showed focal confluent necrosis of muscle fibres with inflammatory cell infiltration, edema with fibroblastic proliferation and myophagocytosis along with extravasation of red blood cells. In the tetramethylpyrazine phosphate tablets + ISO groups, there was less edema and myonecrosis with less inflammatory cells. 

## 3. Discussion

Elevated left ventricular end-diastolic pressure has been described as a risk factor in cardiac surgery [23]. The rate of left ventricular pressure development (dP/dt) has been widely used to evaluate myocardium contractility. In the present study, the effects of TPT treatment in a rat model of failure were examined. This study shows that TPT treatment results in an improved cardiac function, as shown by a significant decrease of LVEDP and a significant increase in LVSP, +dp/dtmax, and −dp/dtmax. Furthermore, our data indicate that TPT restores LVMI and RVMI to sham levels and decreases the Ang II, cholesterol, ANP and endothelin levels, indicating that TPT treatment may improve hemodynamics, decrease ventricular hypertrophy, inhibit myocardial fibrosis and excessive activation of neurohumoral factors. Decreased serum Ang II, cholesterol, ANP and endothelin levels might be one of mechanisms whereby TPT delays heart failure development.

Tumor necrosis factor (TNF)-α, interleukin (IL)-6, and other inflammatory cytokines have been recognized as correlates of heart failure disease. Prolonged inflammatory reaction is considered part of the pathogenesis of a variety of cardiac diseases, including heart failure and adverse left ventricular (LV) remodeling [24]. This is supported by the finding that levels of proinflammatory cytokines, including tumor necrosis factor (TNF)-α, interleukin (IL)-6 and IL-1β, increase during heart failure [25]. In fact, elevated circulating levels of TNF-α and IL-6 have been reported as independent predictors of mortality in patients with heart failure [26,27,28]. Excessive TNF-α expression can inhibit sarcoplasmic reticulum Ca^2+^ pump, and result in intracellular Ca^2+^ imbalance. In addition, high TNF-α expression can still decrease sensitive of NO to Ca^2+^. As a result, this affect myocardial contractility [29,30,31,32]. TNF-α can promote expression and release of cytokine, e.g., IL-1β, IL-6, IFN-α *et al.* This will cause an indirect negative inotropic effect, and further strengthen negative inotropic effect of TNF-α [33]. TNF-α can still induced cardiomyocyte apoptosis [34]. In the present study, TPT treatment may decrease plasma TNF-α, and IL-6 levels, indicating that TPT delay heart failure by inhibiting TNF-α, and IL-6 secretion, and improving immunity system, and strengthen myocardial contractility.

As shown in Table 5, plasma levels of all compounds measured were significantly lower in rats compared to controls, with the exception of MDA, which was significantly higher in rats compared to controls (*p* < 0.01). In animal models of heart failure, lower levels and activities of antioxidants have been described [35,36,37,38]. Antioxidants might be depleted in heart failure due to increased ROS scavenging [39,40,41], or be already deficient due to lifestyle, disease, or age-related reasons and facilitate the worsening of oxidative stress [41,42,43]. Increased ROS production has been shown to be involved in myocardial apoptosis [44,45] and remodeling [46]. CAT has been shown to be more effective at higher H_2_O_2_ concentrations than GSH-Px [47]. GSH-Px requires glutathione as a co-substrate for its enzymatic activity whereas CAT enables the cell to decompose hydrogen peroxide regardless of the cellular concentration of glutathione. It has been shown that under chronic oxidative stress by direct challenge of myocytes with hydrogen peroxide, CAT but not GSH-Px was selectively induced by transcriptional activation [48,49]. It has been reported that increased oxidative stress in human end-stage heart failure results in a specific upregulation of CAT gene expression [50]. In the present study, TPT treatment can significantly decrease myocardium MDA level and increase GSH level, SOD, CAT and GSH-Px activities in heart failure rats. This indicates that TPT delay heart failure partly by decreasing lipid peroxidation level and enhancing antioxidant enzymes activities.

## 4. Experimental

### 4.1. Materials

Tetramethylpyrazine phosphate tablets were purchased from Beijing Yangjiao Medicine Ltd.

### 4.2. Animal

Two-month-old Wistar rats weighing 140–100 g, purchased from the Experimental Animal Center of Shandong University (Jinan, China), were acclimatized for seven days to the animal house, and maintained at standard conditions of temperature and relative humidity, with a 12 h light/12 h dark cycle. Water and standard pellet diet were provided *ad libitum*. The current work was carried out after approval by Shandong University Institutional Animal Ethical Committee.

### 4.3. Experimental Design

Heart failure in rats was induced by ISO (2 mg/kg·days) by peritoneal injection for three days according to the procedure described previously. Then, surviving 41 rats were divided into four groups and received oral administration of tetramethylpyrazine phosphate tablets 2 mg/kg (group II, n = 10), 3 mg/kg (group III, n = 11) and 4 mg/kg (group IV, n = 10) every day, respectively, or vehicle (physiological saline) (group V, n = 10) for 25 days. Eight-week-old wistar rats without receiving ISO were used as normal control group (I) (n = 11). The dose used in the experiments was determined on the basis of the our pre-experiment.

During ISO treatment, three rats died in the ISO model group; three rats (survival rate 23%) died in the TPT+ISO (2 mg/kg) group; two rats (survival rate 15%) died in the TPT+ISO (3 mg/kg) group; three rats (survival rate 23%) died in the TPT+ISO (4 mg/kg) group. Overall, 79% of the injected rats survived for the subsequent experiments. During the next one month, two rats (one for ISO model group (mortality rate 10%); one for TPT+ISO (2 mg/kg) group (mortality rate 10%) died. 


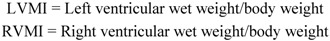


### 4.4. Hemodynamic Assessment

Hemodynamic measurements were performed. In brief, the right carotid artery was exposed and a catheter tip micromanometer (SPR 407, Millar Instruments, Houston, TX, USA) was introduced into the cavity of the left ventricle through the carotid artery. LVSP, LVEDP, +dP/dtmax and −dP/dtmax were measured by means of a pressure transducer (Model SPR-838, Millar Instruments) and a differentiator, respectively.

### 4.5. Biochemical Analysis

Ang II, cholesterol, ANP and endothelin levels were measured using nuclear radioimmunoassay kits. The levels of TNF-α and IL-6 were measured in serum samples using a commercially available ELISA kit for rat TNF-α and IL-6. All procedures were performed according to the kit instructions.

Lipid peroxide levels, expressed in terms of MDA, were determined according to the method of Buege and Aust [51]. GSH was measured according to the method of Ellman [52]. Superoxide dismutase (SOD) was assayed according to Misra and Fridovich [53]. Glutathione peroxidase (GSH-Px) activity assayed using the method of Chiu *et al.* [54]. Catalase (CAT) activity was determined after 30 min preincubation of the postmitochondrial fraction of the heart homogenate with 1% Triton X-100 by measuring the decrease in absorbance of hydrogen peroxide at 240 nm [55]. 

### 4.6. Histopathological Examination

Myocardial tissues from all the groups were subjected to histopathological studies by the following method of Luna [56]. The tissues were fixed in formalin (10%), routinely processed and embedded in paraffin wax. Paraffin section (5 μm) were cut on glass slides and stained with hematoxylin and eosin (H&E) after dewaxing, and examined under a light microscope.

### 4.7. Statistical Analysis

Data obtained in the current study are expressed as mean ± SD. A computer program (SPSS 9.0, SPSS Inc. Chicago, IL, USA) was used for statistical analysis. The one-way ANOVA and post hoc multiple comparison tests (LSD) were performed on the biochemical variable data to examine the differences among the groups. A *p* value of <0.05 was considered to be statistically significant.

## 5. Conclusion

Our present shows that TPT can improve the immunity system, strengthen myocardial contractility, decrease lipid peroxidation, and enhance antioxidant enzyme activities in heart failure rats.
